# Zukunftsperspektive in der Chirurgie aus deutscher Sicht

**DOI:** 10.1007/s00104-022-01793-7

**Published:** 2023-01-02

**Authors:** Christiane J. Bruns

**Affiliations:** Klinik und Poliklinik für Allgemein‑, Viszeral‑, Tumor- und Transplantationschirurgie, Universitätsklinikum Köln (AöR), Kerpener Str. 62, 50937 Köln, Deutschland

**Keywords:** Gesundheitspolitik, Mindestmengen, Zentralisierung, Personalisierte Präzisionsbehandlung, Medizintechnik, Healthcare policy, Minimum quantity, Centralization, Personalized precision treatment, Medical technology

## Abstract

In diesem Beitrag werden zum Thema „Zukunftsperspektiven in der Chirurgie aus deutscher Sicht“ zwei Aspekte diskutiert: zum einen gesundheitspolitische Themen wie die anstehenden Veränderungen des Gesundheitssystems, wie geforderte Mindestmengen mit konsekutiver Zentralisierung sowie die zunehmende Vielfalt unserer Bevölkerung mit Chancen und Herausforderungen für die Chirurgie, zum anderen die flächendeckende personalisierte Präzisionsbehandlung, die individualisierte Organtransplantation und visionäre Entwicklungen in der Medizintechnik mit künstlicher Intelligenz.

## Abstract (en)

In this article two aspects of the topic of future perspectives in surgery from a German point of view are discussed: firstly, topics of healthcare policy, such as upcoming alterations of the healthcare system, including required minimum quantities with subsequent centralization and the increasing diversity of our population with chances and challenges for surgery. Secondly, comprehensive personalized precision treatment, individualized organ transplantation and visionary developments in medical technology with artificial intelligence.

## Gesundheitspolitische Themen

### Mindestmengen

Bereits vor 10 Jahren haben Birkmeyer et al. im *New England Journal of Medicine and Surgery* publiziert, dass Krankenhausvolumen und chirurgische Mortalität für komplexe Eingriffe wie beispielsweise die Ösophagus- und die Pankreaschirurgie direkt miteinander korrelieren [1]. Auch wurde festgestellt, dass unabhängig vom Krankenhausvolumen die Mortalität bei Low-volume-Chirurgen höher ist als bei High-volume-Chirurgen.

Ebenso Aufsehen erregt hat die Publikation von Nimptsch et al. in der Fachzeitschrift *Deutsches Ärzteblatt* 2015 [2]. Hier wurden in 400 deutschen Krankenhäusern Fallzahlen, Krankenhaussterblichkeit und Komplikationsmanagement in der komplexen Ösophaguschirurgie untersucht und dabei festgestellt, dass die jährliche mediane Leistungsmenge dieser Krankenhäuser genau bei 6 Fällen lag, was dramatisch wenig ist. Hinzu kommt, dass in 85 % dieser operierten Fälle das komplexe Krankheitsbild Ösophaguskarzinom zugrunde lag. Ebenfalls hat diese Publikation gezeigt, dass die Speiseröhrenchirurgie eine komplikative Chirurgie sein kann, 50 % der Patienten haben irgendeine Art von Komplikation, und zwar unabhängig von der Krankenhausfallzahl.

Allerdings zeigte sich, dass Patienten, die in Krankenhäusern mit sehr geringer Fallzahl behandelt wurden, zu 20 % an ihren Komplikationen verstarben, während in Krankenhäusern mit sehr hoher Fallzahl dieser Anteil bei nur 12 % lag.

Die vom Gemeinsamen Bundesauschuss (G-BA) geforderte Mindestmenge für die komplexe Ösophaguschirurgie beträgt ab 2023 *n* = 26. Diese Zahl entspricht nach der Quintileneinteilung der 400 Krankenhäusern in der Arbeit von Nimptsch et al. einem Krankenhaus mit hoher Fallzahl an komplexer Ösophaguschirurgie, nicht mit sehr hoher Fallzahl, die wäre *n* = 62. Die Analyse zu „failure to rescue“ hat gezeigt, dass erst bei einer sehr hohen Fallzahl – nämlich *n* = 62 und mehr – die Wahrscheinlichkeit, an Komplikationen zu versterben, substanziell gegenüber geringen, mittleren und hohen Fallzahlen sowie signifikant gegenüber sehr geringen Fallzahlen absinkt.

Mindestmengen werden kritisch diskutiert, haben aber bereits als Bewertungsgrundlage in verschiedene nationale Qualitätsprüfungen Einzug gefunden, darüber hinaus sind sie international zum Teil bereits akzeptierte Benchmarks.

In Deutschland werden bereits über mehrere Jahre Mindestmengen in Zertifizierungsprozessen der Deutschen Gesellschaft für Allgemein- und Viszeralchirurgie für Kompetenz‑, Referenz- und Exzellenzzentren in der Speiseröhrenchirurgie, der Pankreaschirurgie etc. gefordert, ebenso für Zertifizierungsprozesse der Deutschen Krebsgesellschaft wie Darm‑, Pankreas‑, Leber- und Ösophaguskrebszentren.

Als Ergebnis einer großen europäischen Multicenterstudie konnte bereits 2015 von Markar et al. gezeigt werden, dass sich die Qualitätsindikatoren nach Speiseröhrenchirurgie wie 30-Tage-Mortalität oder Hospitalmortalität mit den dazugehörigen Ursachen wie Anastomosenleckage oder pulmonalen Komplikationen erst signifikant ab einer Fallzahl von* n* = 80 und mehr verbessern, dementsprechend ist die mit 26 vom G‑BA gewählte Mindestmengenfallzahl sogar eher niedrig [3].

Zusammengefasst bleiben Mindestmengen und konsekutive Zentralisierung ein gesundheitspolitisches Spannungsfeld. Die sog. „Composite-Qualitätsindikatoren“ wie beispielsweise „failure to rescue“ sind mittelfristig sicherlich zur Festlegung einer Struktur- und Prozessqualität wichtiger als Mindestmengen.

Dennoch ist die Festlegung von Mindestmengen für komplexe chirurgische Eingriffe und seltene Erkrankungen wichtig. Ob es die erste und alleinige Maßnahme zur Umstrukturierung der Krankenhauslandschaft sein sollte, bleibt zu diskutieren.

Mindestmengen führen natürlich zur Zentralisierung und zur Veränderung der Krankenhauslandschaft. Das führt im weiteren Verlauf natürlich an Standorten, die die Mindestmengen – beispielsweise zur Ösophagus- und Pankreaschirurgie – nicht mehr erfüllen, zum Verlust der Weiterbildungsmöglichkeit zur speziellen Viszeralchirurgie. Damit verliert dieser Standort Attraktivität und auf Dauer gegebenenfalls auch ärztliche Fachkräfte. Entsprechend müssen zukünftig auch Aus- und Weiterbildungskonzepte neu entwickelt werden. Das könnte beispielsweise durch gezielte Kooperationen mit Partnerkliniken, die Mindestmengen erfüllen, mit Mitarbeiteraustausch, gemeinsamen Fort- und Weiterbildungen sowie modernen Vertragsstrukturen umgesetzt werden.

Ebenfalls zu überdenken ist die teilweise unzureichende Infrastruktur (Operationskapazität, Intensivkapazität, Pflegekräftebedarf) der bereits existierenden High-volume-Zentren für Mindestmengeneingriffe, die das zusätzliche Aufkommen beispielsweise der Ösophagus- und Pankreaschirurgie kompensieren sollen.

### Diversität der Bevölkerung

Ein weiteres gesundheitspolitisches Thema – auch sehr wichtig für die Chirurgie – ist die zunehmende Vielfalt in der Bevölkerung. In Deutschland leben 82 Mio. Menschen, 19,3 Mio. Menschen haben einen Migrationshintergrund, 10,8 Mio. Menschen schwere Behinderungen und es existieren über 400 religiöse Gemeinschaften. Das heißt, es bestehen Diversitäten in nationaler und ethnischer Herkunft, Hautfarbe, Religion, Geschlecht, Behinderung, Alter und in vielerlei anderer Hinsicht. Diversität muss berücksichtigt werden in der medizinischen Versorgung, der Patientenbetreuung, Mitarbeiterführung bis hin zur Interpretation von Forschungsergebnissen und ihrer klinischen Umsetzung, da viele Forschungsergebnisse eigentlich nur an männlichen Kollektiven erhoben wurden.

Seit 2018 ist in der American Surgical Association (ASA) der Aspekt „diversity“ proaktiv aufgegriffen worden, da man festgestellt hatte, dass mit steigender Diversität und Gleichbehandlung in der Chirurgie mehr talentierte akademische Mediziner rekrutiert werden konnten und damit letztendlich auch die Gesundheit der Gesellschaft verbessert wurde. 2019 wurden die Ergebnisse einer Metaanalyse zur Evaluation des Einflusses von Vielfalt und Innovation auf Gesundheitsergebnisse in den USA publiziert. Es wurden insgesamt 16 Studien untersucht, und es konnte ein positiver Effekt von Vielfalt und Innovationskraft auf die finanzielle Leistungsfähigkeit einer klinischen Abteilung festgestellt werden. Ferner zeigten sich eine Steigerung des Wohlempfindens der Patienten und eine Verbesserung der Kommunikationsfähigkeit und Risikobewertung eines chirurgischen Teams.

Wie sieht „diversity“ in Deutschland aus? Diversität wird in Deutschland gleichgesetzt mit „Gender“. Das ist auch nach wie vor ein großes Thema. Von den insgesamt 35.000 Chirurginnen und Chirurgen sind gerade 19 % weibliche chirurgische Kräfte. Das ist deutlich zu wenig, insbesondere wenn man das prozentuale Verhältnis der Chirurginnen zum Anteil an Ärztinnen in anderen Fachgesellschaften sieht. Sehr wenige Frauen sind in Führungspositionen in der Medizin, noch weniger in der Chirurgie. Führung wird nach wie vor gemeinhin mit männlichen stereotypen Verhaltensweisen verbunden, teilweise sogar unbewusst. Das muss sich in der Zukunft ändern!

Betrachtet man den Einfluss von Diversität in der Patientenversorgung, sei auf eine aktuelle Publikation aus dem Jahr 2022 zu den klinischen Ergebnissen in der chirurgischen Behandlung gastrointestinaler Tumorerkrankungen in den USA unter Berücksichtigung der Diversität der Patienten verwiesen. Hier zeigte sich, das die R0-Resektionsrate am Absetzungsrand großer onkologischer Operationen bei kaukasischen Patienten wesentlich höher war als bei afroamerikanischen Patienten, entsprechend lag eine adäquate Lymphknotendissektion wesentlich häufiger bei kaukasischen als bei afroamerikanischen Patienten vor. Auch hatten kaukasische Patienten wesentlich häufiger Zugang zu neoadjuvanter oder palliativer Chemo- oder Radiochemotherapie als afroamerikanische Patienten.

Auch in Deutschland wird zukünftig Diversität vielschichtiger zu diskutieren sein – Vielfältigkeit ist eine großartige Chance, bleibt immer eine Herausforderung und sollte nicht als Problem betrachtet werden.

Von den 370.000 berufstätigen Ärztinnen und Ärzten sind bereits 15 % ausländische Fachkräfte aus Syrien, Rumänien, Griechenland, Österreich, der russischen Föderationen und natürlich den europäischen Nachbarstaaten. Nicht zuletzt um den bereits absehbaren Bedarf an ärztlichen Fachkräften in Deutschland auszubilden, damit auch zukünftig die chirurgisch-medizinische Versorgung aller Menschen in Deutschland gesichert ist, ist zukünftig viel mehr Diversität notwendig.

## Chirurgisch-technische Themen

Ein weiteres Thema zu „Zukunftsperspektiven in der Chirurgie“ ist die in zunehmende personalisierte Präzisionsbehandlung in der onkologischen Chirurgie. Warum ist das für Patienten mit Tumorerkrankung so wichtig? Jeder Patient ist individuell hinsichtlich des biologischen Alters, der assoziierten Komorbidität, des klinischen Ausmaßes der Tumorerkrankung und des persönlichen Behandlungswunsches, insbesondere aber hinsichtlich der individuellen Tumorbiologie und Molekularpathologie der Erkrankung. Entsprechend muss die chirurgische Therapie bezüglich Indikationsstellung, erforderlicher chirurgisch-onkologischer Radikalität und funktioneller Machbarkeit angepasst werden. Ganz wichtig dabei sind der gewählte Zeitpunkt der Chirurgie im multimodalen Behandlungskonzept sowie die Lebensqualität nach der chirurgischen Behandlung.

Die personalisierte Behandlung beim Vorliegen eines Pankreaskarzinoms könnte in Zukunft entsprechend der individuellen Tumorbiologie so aussehen: Beim klinischen Verdacht einer Pankreasraumforderung erfolgt zunächst ein bildgestütztes multiregionales Tumorsampling sämtlicher Läsionen (Primärtumor und Metastasen) inklusive sog. „liquid biopsies“ mit anschließender zum einen klassischer histopathologischer Aufarbeitung, zum anderen „high-throughput multiomics in-situ tumor profiling“. Sämtliche Daten des Patienten werden gesammelt, dann mithilfe künstlicher Intelligenz (KI) und Systembiologie ausgewertet, um daraus ein individualisiertes Behandlungskonzept oder personalisiertes Disease-Monitoring zu entwickeln, mit dem einen Ziel, die Erkrankung zu heilen, oder mit dem anderen Ziel, Lebenszeit durch Chronifizierung der Erkrankung zu verlängern. Durch zunehmende Personalisierung des therapeutischen Vorgehens unter Kenntnis der Erkrankungsbiologie wird es zukünftig zur gezielten Ausweitung der Indikation zum operativen Vorgehen beispielsweise bei bereits metastasierten Tumorerkrankungen kommen. Es wurde bereits der Begriff „Oligometastasierung“ bei soliden Tumorerkrankungen geprägt, der sich aktuell lediglich nach bildgebenden und wenigen klinischen Parametern definiert. Zurzeit sollten Oligometastasen nur unter Studienbedingungen operativ oder lokal-therapeutisch behandelt werden.

Eine weitere substanzielle Herausforderung der Zukunft ist die Organtransplantation. Es existiert ein genereller Organmangel, entsprechend steigt die Diskrepanz zwischen Organangeboten und Transplantationsindikationen. Bei nach wie vor unzureichenden Alternativtherapien wird das Risiko, auf der Transplantationsliste bei Mangel an zeitgerecht vorhandenen Spenderorganen an der Grunderkrankung zu versterben, weiter steigen. Dies trifft besonders für das hepatozelluläre Karzinom als Indikation zur Lebertransplantation zu und bezieht sich auch auf die bislang unzureichenden Bridging-Therapien über die Wartezeit.

Nicht nur gibt es zu wenige Organe gerade für die Lebertransplantation, auch steigt bei den noch vorhandenen Organspenden der Anteil an marginalen Organen, insbesondere verfetteten Organen mit steigender Übergewichtigkeit der Bevölkerung insbesondere in den USA, zeitlich etwas verzögert allerdings auch in Europa. Gerade bei marginalen Organen in der Lebertransplantation ist die Ischämiezeit – und damit Transportzeit – kritisch. Ganz entscheidend für die Zukunft in der Organtransplantation wird auch der zunehmende Mangel an Fachkräften mit chirurgischer Transplantationsexpertise.

Wie könnte die Transplantationsmedizin in der Zukunft aussehen?

Hier spielt die Xenotransplantation für Herz, Pankreasinseln, evtl. auch Niere durch die genetische Modifikation von Xeno-Organen (Schwein/„non-human-primates“) durch „crispr/cas‑9 gene editing“ (GalSafe) sowie die Modulation der Alloimmunantwort durch siRNA-Therapie bzw. Genmodulation durch Antisense-Oligonuklitide (ASOs) kombiniert mit Maschinenperfusion sicher eine entscheidende Rolle.

Zukünftig wird die Maschinenperfusion – und zwar die normotherme Maschinenperfusion – eine dynamische Plattform für regenerative Prozesse marginaler Organe sein. Dazu zählen „Defatting-Strategien“, Senolytikagabe, Stammzell- oder Progenitorgaben oder die oben genannte Modulation der Alloimmunantwort. Des Weiteren werden sich möglicherweise Zelltherapien zur individualisierten Immunsuppression wie CAR-Tregs und BAR-Tregs („chimeric antigen receptor Tregs“, „B-cell antibody receptor T cells“) durchsetzen.

Ein weiteres Schlagwort für die Zukunft der Organtransplantation ist die „Transplantationsonkologie“. Lebertransplantation bei biologisch und klinisch stabiler, diffuser kolorektaler Lebermetastasierung hat sich in Skandinavien, Frankreich, Italien und Kanada über verschiedene Studienprotokolle durchgesetzt. Ähnliche Konzepte werden in Deutschland in kombinierten Verfahren unter Nutzung der ALPPS(„associating liver partition and portal vein ligation for staged hepatectomy“)-Prozedur und der auxilliären linkslateralen Lebertransplantation nun begonnen.

Abschließend sei die Entwicklungen in der Medizintechnik sowie der künstlichen Intelligenz und ihr zukünftiger Stellenwert in der Chirurgie erwähnt. Historisch begann die Entwicklung von Operationsrobotern ungefähr 1980 mit dem Probot-Roboter zur transurethralen Prostatektomie oder dem Robodoc zur Arthroskopie des Hüftgelenkes. Dominiert hat die operative Medizin dann schließlich von 1998 bis aktuell die Entwicklung des ZEUS-Systems und des Da-Vinci-Roboters. Weitere Operationsrobotersysteme werden aktuell hergestellt. Es gibt zurzeit bereits Geräte zur autonomen Navigation für die Implantation von Herzklappen sowie zur autonomen Herstellung von Darmanastomosen am Schweinedarm. Die Übertragung dieser Technologie auf den Menschen ist absehbar.

Sicher wird man in der Zukunft auch von „KI-assistierter Chirurgie“ sprechen. Operationsrisikoeinschätzungen, Komplikationsrisiko, Pathologie an Gefäßen wie Stenosen an großen Stammgefäßen oder anatomische Gefäßvarianten werden über neuronale Netze ausgewertet, um daraus Operationsrisikoabwägung, Operationsstrategieentscheidungen sowie Komplikationsmanagement vorherzusagen.

Für die chirurgische Weiterbildung wird möglicherweise die maschinelle Auswertung von Bilddaten sowie Bewertung durch KI die Zukunft sein. So wie es einen Tesla-Autopiloten gibt, wird es zukünftig den durch KI berechneten chirurgischen Autopiloten mit farblich markierten „Go-and-no-go-Zonen“ – beispielsweise für einen der häufigsten Eingriffe wie der laparoskopische Cholezystektomie – geben, der zwar ein klassischer Standardausbildungseingriff ist, allerdings bei Komplikationen fatale Folgen haben kann.

## Fazit

Zusammenfassend wird die Zukunft der Chirurgie in Deutschland gesundheitspolitisch geprägt sein durch Zentrumsbildung mit Qualitätssteigerung basierend auf Qualitätsindikatoren zur Strukturverbesserung und Mindestmengen. Es werden entsprechend innovative Netzwerke mit Kooperationspartnern zur Aus- und Weiterbildung und Nachwuchsförderung entstehen müssen. Die steigende Vielfalt unserer Bevölkerung sollte auch in der Chirurgie als Chance gesehen werden. Modernste Technologie im Operationssaal unterstützt durch KI und innovatives Imaging müssen weiterentwickelt werden. Künstliche Intelligenz kann ein entscheidender Teil der chirurgischen Qualitätssicherung und ein nützliches Tool in der chirurgischen Aus- und Weiterbildung sein.

Inhaltlich wird sich auch die Chirurgie in allen Facetten zunehmend in Richtung Therapiepersonalisierung entwickeln, was unter anderem zu maßgeschneiderter Erweiterung von Operationsindikationen führen kann. Entscheidend bleibt dabei in allen chirurgischen Bereichen das Verständnis für die Biologie der zugrunde liegenden Erkrankungen.
